# Comparative analysis of thylakoid protein complexes in state transition mutants *nsi* and *stn7*: focus on PSI and LHCII

**DOI:** 10.1007/s11120-020-00711-4

**Published:** 2020-01-23

**Authors:** Minna M. Koskela, Annika Brünje, Aiste Ivanauskaite, Laura S. Lopez, Dominik Schneider, Rachael A. DeTar, Hans-Henning Kunz, Iris Finkemeier, Paula Mulo

**Affiliations:** 1 Department of Biochemistry, Molecular Plant Biology, University of Turku, Biocity A, Tykistökatu 6, 20520 Turku, Finland; 2 Plant Physiology, Institute of Plant Biology and Biotechnology, University of Münster, Schlossplatz 7, 48149 Münster, Germany; 3 Plant Physiology, School of Biological Sciences, Washington State University, Pullman, WA 99164-4236 USA; 4 Compact Plants Phenomics Center, Washington State University, Pullman, WA 99164 USA; 5 Institute of Biological Chemistry, Washington State University, Pullman, WA 99164-6340 USA; 6 Present Address: Centre Algatech, Institute of Microbiology, Czech Academy of Sciences, Novohradská 237 - Opatovický mlýn, 379 81 Třebon, Czech Republic

**Keywords:** Arabidopsis, Light-harvesting complex, Lysine acetylation, State transitions, Photosystem I

## Abstract

The photosynthetic machinery of plants can acclimate to changes in light conditions by balancing light-harvesting between the two photosystems (PS). This acclimation response is induced by the change in the redox state of the plastoquinone pool, which triggers state transitions through activation of the STN7 kinase and subsequent phosphorylation of light-harvesting complex II (LHCII) proteins. Phosphorylation of LHCII results in its association with PSI (state 2), whereas dephosphorylation restores energy allocation to PSII (state 1). In addition to state transition regulation by phosphorylation, we have recently discovered that plants lacking the chloroplast acetyltransferase NSI are also locked in state 1, even though they possess normal LHCII phosphorylation. This defect may result from decreased lysine acetylation of several chloroplast proteins. Here, we compared the composition of wild type (wt), *stn7* and *nsi* thylakoid protein complexes involved in state transitions separated by Blue Native gel electrophoresis. Protein complex composition and relative protein abundances were determined by LC–MS/MS analyses using iBAQ quantification. We show that despite obvious mechanistic differences leading to defects in state transitions, no major differences were detected in the composition of PSI and LHCII between the mutants. Moreover, both *stn7* and *nsi* plants show retarded growth and decreased PSII capacity under fluctuating light as compared to wt, while the induction of non-photochemical quenching under fluctuating light was much lower in both *nsi* mutants than in *stn7*.

**Electronic supplementary material:**

The online version of this article (10.1007/s11120-020-00711-4) contains supplementary material, which is available to authorized users.

## Introduction

Light is an important environmental signal affecting most aspects of plant life including germination, phototropism and flowering. Additionally, light is the driving force of photosynthetic electron transfer and carbon assimilation, which provide plants with the energy needed for their growth and development. The light-harvesting complexes (LHCs), composed of chlorophyll and other pigment molecules bound to the thylakoid-embedded LHC proteins, absorb light energy and funnel it towards the reaction centers P680 in Photosystem II (PSII) and P700 in Photosystem I (PSI). Excitation of the special pair of reaction center chlorophylls is followed by charge separation, splitting of water and electron transfer through the redox-active components of the thylakoid membrane, which finally leads to the reduction of NADP^+^ to NADPH. The electron transfer is accompanied by proton translocation into the thylakoid lumen, and the resulting proton gradient is harnessed by the ATP synthase for ATP production. Subsequently, NADPH and ATP are used in the Calvin–Benson cycle for CO_2_ assimilation. Importantly, several enzymes functioning in carbon assimilation are also redox activated according to ambient illumination (Buchanan and Balmer 2005).

Plants have developed several systems to sense changes in ambient illumination, such as the red and blue light-sensing light receptor molecules, phytochromes and phototropins, respectively. However, the photosynthetic machinery can sense changes in light quality and intensity also directly via the redox state of the plastoquinone (PQ) pool, which triggers appropriate long- and short-term responses for balancing the redox poise between the two photosystems. The redox state of the PQ pool is known to regulate gene expression both in the chloroplasts and the nucleus, which results in stoichiometry adjustments of the photosystems to meet the needs of the plant under new environmental conditions in the long term (Steiner et al. 2009; Puthiyaveetil et al. 2010, 2012). Short-term acclimation is achieved through thermal dissipation of absorbed excess energy (non-photochemical quenching (NPQ)) in seconds, and via state transitions, i.e. re-equilibration of the absorption cross-section of PSII and PSI within the time scale of minutes (Bonaventura and Myers 1969; Murata 1969). LHCII is an important player in these acclimation processes, as it functions both in harvesting and dissipation of light energy depending on ambient conditions (Rochaix 2014). State transitions are mediated through the phosphorylation and dephosphorylation of LHCII proteins: phosphorylation of LHCII results in association of LHCII to PSI (state 2), while LHCII dephosphorylation leads to reallocation of energy to PSII (state 1) (Bennett 1977; Pietrzykowska et al. 2014). Phosphorylation of the major LHCII antenna proteins, LHCB1 and LHCB2, is catalyzed by the STN7 kinase, which is activated upon docking of PQH_2_ to the Q_o_ site of the Cyt b_6_f complex (Bennett 1977; Allen et al. 1981; Depège et al. 2003; Bellafiore et al. 2005). On the other hand, over-excitation of PSI results in the oxidation of the PQ pool, deactivation of STN7 and dephosphorylation of LHCII by the TAP38/PPH1 phosphatase (Pribil et al. 2010; Shapiguzov et al. 2010).

The composition and phosphorylation patterns of light-harvesting complexes under various conditions have been well studied in numerous phototrophic organisms. In *Arabidopsis thaliana* (hereafter Arabidopsis), LHCII is composed of LHCB1-LHCB6 proteins (Jansson 1999). LHCB1-LHCB3 form the trimeric LHCII antenna, which may be either strongly (S-LHCII composed of LHCB1 and LHCB2), moderately (M-LHCII composed of LHCB1 and LHCB3), or loosely (L-LHCII composed of mainly LHCB1 and LHCB2) bound to PSII. The S-trimer is bound to the PSII core monomer via minor antenna protein LHCB5, whereas the M-trimer is associated to the core via LHCB4 and LHCB6 proteins. Due to the very loose interaction, the binding site of the L-trimer is still elusive (Dekker and Boekema 2005; Kouril et al. 2005; Galka et al. 2012). Approximately, half of the LHC protein pool at the thylakoids form the so-called “extra” or “free” L-LHCII trimers, which are not tightly bound to the photosystems, but serve as a shared antenna for both PSII and PSI (Grieco et al. 2012; Wientjes et al. 2013). LHCI, in turn, is composed of LHCA1–LHCA4 proteins, which are surrounding PSI in the shape of a half-moon (Boekema et al. 2001; Ben-Shem et al. 2003). Upon phosphorylation of LHCB2 protein, a portion of L-LHCII becomes associated with PSI either through the PSAH/PSAI/PSAL/PSAO proteins and/or through the LHCA antenna (Lunde et al. 2000; Galka et al. 2012; Crepin and Caffarri 2015; Longoni et al. 2015; Benson et al. 2015; Pan et al. 2018).

We have recently identified Arabidopsis mutant lines devoid of the chloroplast lysine acetyltransferase NSI, which similar to the *stn7* loss-of-function mutant, are not capable of forming the PSI–LHCII complex and thus performing state transitions (Depège et al. 2003; Bellafiore et al. 2005; Koskela et al. 2018). Intriguingly, no differences in the pattern of LHCII phosphorylation were detected between the *nsi* and wt plants, but the *nsi* plants possessed decreased lysine acetylation of several chloroplast proteins as compared to wt (Koskela et al. 2018). Hence, the mechanisms leading to defects in state transitions in the *stn7* and *nsi* mutants must be fundamentally different. To get a deeper insight into these mechanistic differences, we compared the composition of the protein complexes involved in state transitions (i.e. PSI complex and LHCII trimers) using large pore (lp) Blue Native (BN) gel electrophoresis for protein complex separation and LC–MS/MS for analyzing specific protein complexes excised from the gel. Moreover, we have compared the growth and photosynthetic performance of *stn7* and *nsi* plants with fluctuating light treatment, which has previously been shown to have a strong effect on the growth of *stn7* (Bellafiore et al. 2005; Tikkanen et al. 2010).

## Material and methods

### Plant material

For lpBN gel electrophoresis and subsequent MS analysis, *Arabidopsis thaliana* wt plants (Col-0) and the T-DNA mutant lines *nsi-1* (SALK_033944), *nsi-2* (SALK_020577) (Koskela et al. 2018) and *stn7* (SALK_073254) (Tikkanen et al. 2006) were grown in 8 h light/16 h darkness at photosynthetic photon flux density (PPFD) of 100 µmol m^−2^ s^−1^, 50% humidity and + 23 °C. For physiological experiments, plants were grown in 12 h light/12 h darkness at PPFD of 90 µmol photons m^−2^ s^−1^ for 2 weeks followed by a shift to fluctuating light (cycles of 4 min 90 µmol m^−2^ s^−1^ followed by 1 min 900 µmol m^−2^ s^−1^) for 2 weeks (Schneider et al. 2019).

### Growth curves and fluorescence measurements

Photosynthesis-related parameters and growth curves were determined with daily induction curves on dark-adapted plants using a MAXI version IMAGING-PAM (IMAG-K7 by Walz, Effeltrich, Germany). Plants were dark-adapted for 20 min before the induction curve was measured for 300 s at 186 µmol m^−2^ s^−^1 with saturating pulses given every 20 s. The reported Y(II), NPQ and Y(NO) values were calculated by averaging the last two data points of the induction curve. Data analysis of the 15-day light-treatment experiment were carried out using the ImagingPAMProcessing toolkit (Schneider et al. 2019).

### Determination of chlorophyll content

Chlorophyll determination was done as described in (Porra et al. 1989). In brief, rosette tissue from plants grown as described above for physiological experiments was harvested individually at 4.5 weeks of age. Tissue was ground in liquid N_2_, and chlorophyll was extracted by incubating 10–20 mg of material in 1.5 mL of ice cold 80% acetone for 2 h on ice. Samples were spun at 15,000 rpm for 5 min, then supernatant was measured in a spectrophotometer at 646, 663 and 750 nm. For statistical analysis, a Shapiro–Wilk test was used to determine if data was normally distributed. If data had a normal distribution, a 2-way ANOVA and a series of Tukey’s multiple comparison tests were used to determine if means were statistically different. If data was not normally distributed, a Kruskal–Wallis test with a series of Dunn’s multiple comparison tests were used to determine if means were statistically different.

The chlorophyll concentration of intact thylakoids, or thylakoids solubilized with digitonin or DM (Järvi et al. 2011) was determined as described previously (Porra et al. 1989).

### Extraction of thylakoid proteins

Fresh Arabidopsis leaves were ground in 300 mM sucrose, 50 mM HEPES–KOH pH 7.6, 5 mM MgCl_2_, 1 mM Na-EDTA, 1.25% BSA, 22 mM ascorbate, and 10 mM NaF. The homogenate was filtered through pre-soaked Miracloth (Millipore) and the filtrate was centrifuged for 4 min, 4000×*g*, + 4 °C. Chloroplasts were lysed by resuspending the pellet in a hypotonic lysis buffer (5 mM sucrose, 10 mM Hepes–KOH pH 7.6, 5 mM MgCl_2_, 10 mM NaF, Pierce™ protease inhibitor (Thermo Scientific)). The lysate was centrifuged at 18,000×*g* for 5 min, + 4 °C, and the pellet was resuspended in 100 mM sucrose, 10 mM Hepes–KoH pH 7.6, 10 mM MgCl_2_, and 10 mM NaF for storage in − 80 °C. Different biological replicates were prepared from plants grown at different times on separate trays.

### Native and 2D gel electrophoresis, trypsin-digestion, LC–MS/MS and data analysis

LpBN gels and samples were prepared as previously described (Järvi et al. 2011). Protein complexes were excised from the lpBN gels, trypsin digested (Morgan et al. 2008), and analyzed using LC–MS/MS as described in (Koskela et al. 2018). The raw spectrum files of all replicates from the individual protein complexes were processed together using the MaxQuant software version 1.5.2.8 (https://www.maxquant.org/) (Cox and Mann 2008; Tyanova et al. 2016), with match between runs and intensity-based absolute quantification (iBAQ) enabled, and peptide and protein FDR < 1%. The spectrum files were searched against the Araport 11 Arabidopsis protein fasta database, a reverse decoy database, and a standard contaminant list integrated into MaxQuant. Lysine acetylation, protein N-term acetylation and methionine oxidation were searched as variable modifications. The resulting ‘protein groups’ data files were further processed with Perseus version 1.6.1.3 (Tyanova et al. 2016). Reverse hits and contaminants were removed. iBAQ intensities were log2 transformed. Technical replicates were averaged and protein groups identified in only one replicate of each genotype were filtered out. Data were analyzed from three independent biological replicates. Protein abundances within each complex were reported as iBAQ values, which were normalized to PSAA (Tables 1 and 2) or LHCB3 (Table 3). The spectrum file of the LHCII trimer complex (digitonin solubilization) was re-analyzed from Koskela et al. (2018) (www.plantcell.org, copyright American Society of Plant Biologists).

**Table 1:** Quantitative proteome analysis according to iBAQ values of the proteins identified within the PSI/PSII bands excised from lpBN-PAGE gels

Proteins/protein groups		Subunit of	Genotype			
Locus/loci	Name(s)		wt	*nsi-1*	*nsi-2*	*stn7*
AT4G28750	PSAE-1	PSI	1.58 (± 0.05)	1.46 (± 0.14)	1.49 (± 0.26)	1.61 (± 0.38)
AT4G02770	PSAD-1	PSI	1.38 (± 0.30)	1.52 (± 0.15)	1.42 (± 0.08)	1.39 (± 0.02)
AT1G55670	PSAG	PSI	1.38 (± 0.48)	1.01 (± 0.36)	0.86 (± 0.39)	0.94 (± 0.19)
ATCG01060	PSAC	PSI	1.01 (± 0.39)	1.15 (± 0.36)	1.10 (± 0.26)	1.06 (± 0.51)
ATCG00350	PSAA	PSI	1.00	1.00	1.00	1.00
AT1G31330	PSAF	PSI	0.89 (± 0.20)	0.99 (± 0.12)	0.93 (± 0.11)	0.92 (± 0.18)
AT1G61520	LHCA3	PSI	0.73 (± 0.12)	0.99 (± 0.08)	0.92 (± 0.15)	0.88 (± 0.36)
AT3G54890	LHCA1	PSI	0.61 (± 0.11)	0.64 (± 0.05)	0.61 (± 0.09)	0.66 (± 0.15)
AT1G52230	PSAH-2	PSI	0.58 (± 0.28)	0.56 (± 0.17)	0.67 (± 0.21)	0.78 (± 0.06)
AT3G47470	LHCA4	PSI	0.58 (± 0.19)	0.82 (± 0.09)	0.80 (± 0.03)	0.81 (± 0.33)
ATCG00340	PSAB	PSI	0.50 (± 0.13)	0.63 (± 0.11)	0.59 (± 0.03)	0.62 (± 0.09)
AT4G12800	PSAL	PSI	0.45 (± 0.07)	0.64 (± 0.07)	0.56 (± 0.06)	0.57 (± 0.13)
AT1G30380	PSAK	PSI	0.31 (± 0.03)	0.38 (± 0.01)	0.36 (± 0.03)	0.32 (± 0.17)
AT3G61470; AT5G28450	LHCA2; CAB family	PSI	0.28 (± 0.17)	0.54 (± 0.08)	0.68 (± 0.24)	0.44 (± 0.26)
AT2G20260	PSAE-2	PSI	0.27 (± 0.08)	0.27 (± 0.02)	0.27 (± 0.05)	0.29 (± 0.03)
AT5G64040	PSAN	PSI	0.13 (± 0.04)	0.15 (± 0.02)	0.18 (± 0.04)	0.17 (± 0.09)
AT2G46820	PSAP	PSI	0.03 (± 0.02)	0.04 (± 0.02)	0.03 (± 0.02)	0.03 (± 0.01)
AT1G08380	PSAO	PSI	0.01 (± 0.02)	0.03 (± 0.02)	0.03 (± 0.01)	0.02 (± 0.02)
ATCG00280	CP43 (PSBC)	PSII	0.24 (± 0.09)	0.16 (± 0.04)	0.12 (± 0.01)	0.16 (± 0.04)
AT1G29920; AT1G29910	LHCB1.1; LHCB1.2*	LHCII	0.24 (± 0.06)	0.14 (± 0.05)	0.11 (± 0.04)	0.09 (± 0.04)
ATCG00680	CP47 (PSBB)	PSII	0.20 (± 0.07)	0.14 (± 0.02)	0.10 (± 0.03)	0.14 (± 0.03)
ATCG00270	D2 (PSBD)	PSII	0.15 (± 0.05)	0.10 (± 0.02)	0.08 (± 0.01)	0.12 (± 0.02)
ATCG00020	D1 (PSBA)	PSII	0.10 (± 0.05)	0.07 (± 0.01)	0.05 (± 0.01)	0.06 (± 0.01)
AT4G10340	CP26 (LHCB5)	PSII	0.07 (± 0.02)	0.08 (± 0.01)	0.06 (± 0.02)	0.07 (± 0.01)
AT3G50820	PSBO-2*	PSII	0.07 (± 0.01)	0.04 (± 0.01)	0.04 (± 0.00)	0.06 (± 0.00)
AT3G08940	CP29-2 (LHCB4.2)	PSII	0.07 (± 0.03)	0.04 (± 0.01)	0.04 (± 0.01)	0.04 (± 0.00)
AT1G15820	CP24 (LHCB6)	PSII	0.06 (± 0.02)	0.04 (± 0.00)	0.04 (± 0.01)	0.04 (± 0.00)
AT3G27690; AT2G05070; AT2G05100	LHCB2.4; LHCB2.2; LHCB2.1	LHCII	0.04 (± 0.05)	0.02 (± 0.00)	0.02 (± 0.01)	0.01 (± 0.01)
ATCG00560	PSBL	PSII	0.03 (± 0.02)	0.02 (± 0.01)	0.00 (± 0.00)	0.03 (± 0.01)
AT5G66570	PSBO-1*	PSII	0.03 (± 0.01)	0.01 (± 0.00)	0.01 (± 0.00)	0.02 (± 0.00)
AT5G01530	CP29-1 (LHCB4.1)	PSII	0.02 (± 0.00)	0.02 (± 0.00)	0.01 (± 0.01)	0.03 (± 0.02)
AT2G34420	LHCB1.5	PSII	0.01 (± 0.01)	0.01 (± 0.00)	0.01 (± 0.00)	0.01 (± 0.00)^a^
AT5G54270	LHCB3	LHCII	0.01 (± 0.01)	0.01 (± 0.00)	0.01 (± 0.00)	0.01 (± 0.00)

Thylakoid membranes were solubilized with digitonin, complexes separated using lpBN-PAGE and selected bands excised for LC–MS/MS analysis. Annotated PSI, PSII and LHC subunits are presented. The quantity of each protein was normalized to PSAA, and the relative protein abundances between genotypes were compared using ANOVA/Brown-Forsythe. Post-hoc analysis of significantly different protein groups (ANOVA *p* < 0.05) was performed using Tukey-HSD. The average relative abundances of detected proteins (± standard deviation) are shown (*n* = 3; *N* = 12)

^*^ANOVA *p* < 0.05

^a^*n* = 2

**Table 2:** Quantitative proteome data analysis according to iBAQ values of the proteins identified within the PSI/PSII bands excised from lpBN-PAGE gels

Proteins/protein groups		Subunit of	Genotype			
Locus/loci	Name(s)		wt	*nsi-1*	*nsi-2*	*stn7*
AT4G02770	PSAD-1	PSI	1.73 (± 0.26)	1.71 (± 0.13)	1.70 (± 0.18)	2.08 (± 0.49)
AT4G28750	PSAE-1	PSI	1.56 (± 0.36)	1.37 (± 0.17)	1.48 (± 0.26)	1.81 (± 0.61)
ATCG01060	PSAC	PSI	1.26 (± 0.05)	1.23 (± 0.28)	1.08 (± 0.29)	1.11 (± 0.19)
AT1G55670	PSAG	PSI	1.21 (± 0.12)	1.19 (± 0.15)	1.06 (± 0.08)	1.16 (± 0.21)
ATCG00350	PSAA	PSI	1.00	1.00	1.00	1.00
AT3G47470	LHCA4	PSI	0.98 (± 0.14)	0.85 (± 0.17)	0.75 (± 0.07)	1.03 (± 0.40)
AT1G61520	LHCA3	PSI	0.97 (± 0.26)	0.88 (± 0.29)	0.90 (± 0.24)	1.10 (± 0.41)
AT1G31330	PSAF	PSI	0.96 (± 0.06)	1.10 (± 0.12)	1.17 (± 0.25)	0.92 (± 0.36)
AT3G54890	LHCA1	PSI	0.70 (± 0.16)	0.61 (± 0.14)	0.69 (± 0.07)	0.67 (± 0.14)
ATCG00340	PSAB	PSI	0.66 (± 0.07)	0.62 (± 0.05)	0.65 (± 0.04)	0.66 (± 0.08)
AT1G52230	PSAH-2	PSI	0.61 (± 0.19)	0.59 (± 0.28)	0.69 (± 0.40)	0.66 (± 0.45)
AT3G61470	LHCA2	PSI	0.59 (± 0.11)	0.45 (± 0.16)	0.57 (± 0.16)	0.52 (± 0.11)
AT4G12800	PSAL	PSI	0.59 (± 0.13)	0.53 (± 0.14)	0.53 (± 0.12)	0.61 (± 0.16)
AT1G30380	PSAK	PSI	0.41 (± 0.11)	0.34 (± 0.05)	0.37 (± 0.03)	0.61 (± 0.33)
AT2G20260	PSAE-2	PSI	0.30 (± 0.11)	0.22 (± 0.02)	0.24 (± 0.05)	0.29 (± 0.11)
AT5G64040	PSAN	PSI	0.08 (± 0.04)	0.09 (± 0.05)	0.10 (± 0.05)	0.10 (± 0.05)
AT1G08380	PSAO	PSI	0.02 (± 0.01)	0.01 (± 0.00)	0.03 (± 0.02)	0.02 (± 0.01)
ATCG00680	CP47 (PSBB)	PSII	0.52 (± 0.00)	0.47 (± 0.00)	0.45 (± 0.00)	0.62 (± 0.00)
ATCG00280	CP43 (PSBC)*	PSII	0.49 (± 0.00)	0.39 (± 0.00)	0.34 (± 0.00)	0.53 (± 0.00)
ATCG00270	D2 (PSBD)	PSII	0.37 (± 0.00)	0.28 (± 0.00)	0.26 (± 0.00)	0.29 (± 0.00)
ATCG00020	D1 (PSBA)	PSII	0.27 (± 0.00)	0.23 (± 0.00)	0.20 (± 0.00)	0.26 (± 0.00)
AT5G66570	PSBO-1	PSII	0.12 (± 0.00)	0.10 (± 0.00)	0.10 (± 0.00)	0.15 (± 0.00)
AT1G29930; AT1G29920; AT1G29910	LHCB1.3; LHCB1.1; LHCB1.2	LHCII	0.09 (± 0.00)	0.08 (± 0.00)	0.08 (± 0.00)	0.09 (± 0.00)
AT4G10340	CP26 (LHCB5)	PSII	0.07 (± 0.00)	0.06 (± 0.00)	0.05 (± 0.00)	0.08 (± 0.00)
ATCG00560	PSBL	PSII	0.06 (± 0.00)^a^	0.02 (± 0.00)^a^	0.02 (± 0.00)	0.08 (± 0.00)^a^
AT3G08940	CP29.2 (LHCB4.2)	PSII	0.01 (± 0.00)	0.01 (± 0.00)	0.01 (± 0.00)	0.04 (± 0.00)
AT3G50820	PSBO-2*	PSII	0.01 (± 0.00)	0.01 (± 0.00)	0.01 (± 0.00)	0.02 (± 0.00)
AT2G05070	LHCB2.2	LHCII	0.03 (± 0.00)	0.02 (± 0.00)	0.01 (± 0.00)	0.02 (± 0.00)
AT2G34430	LHCB1.4	LHCII	0.02 (± 0.01)	0.01 (± 0.01)	0.01 (± 0.00)	0.01 (± 0.01)

Thylakoid membranes were solubilized with dodecyl maltoside, complexes separated using lpBN-PAGE and selected bands excised for LC–MS/MS analysis. Annotated PSI, PSII and LHC subunits are presented. The quantity of each protein was normalized to PSAA, and the relative protein abundances between genotypes were compared using ANOVA/Brown-Forsythe. Post-hoc analysis of significantly different protein groups (ANOVA *p* < 0.05) was performed using Tukey-HSD. The average relative abundances of detected proteins (± standard deviation) are shown (*n* = 3; *N* = 12)

^*^ANOVA *p* < 0.05

^a^*n* = 2

**Table 3:** Quantitative proteome data analysis according to iBAQ values of the proteins identified within the LHCII trimer bands excised from lpBN-PAGE gels

Proteins/protein groups		Subunit of	Genotype			
Locus/loci	Name(s)		wt	*nsi-1*	*nsi-2*	*stn7*
AT2G05070	LHCB2.2	LHCII	8.29 (± 0.75)	15.21 (± 10.94)	14.34 (± 6.24)	11.25 (± 1.84)
AT2G34420	LHCB1.5	LHCII	3.08 (± 0.96)	2.34 (± 0.53)	4.17 (± 1.59)	3.27 (± 1.37)
AT5G54270	LHCB3	LHCII	1.00	1.00	1.00	1.00
AT2G34430	LHCB1.4	LHCII	0.43 (± 0.12)	0.58 (± 0.17)	0.82 (± 0.06)	0.78 (± 0.33)
ATCG00680	CP47 (PSBB)	PSII	0.29 (± 0.05)	0.58 (± 0.27)	0.65 (± 0.13)	0.46 (± 0.08)
ATCG00020	D1 (PSBA)	PSII	0.28 (± 0.08)	0.51 (± 0.27)	0.63 (± 0.14)	0.52 (± 0.10)
ATCG00270	D2 (PSBD)	PSII	0.26 (± 0.04)	0.65 (± 0.28)	0.75 (± 0.20)	0.58 (± 0.11)
AT1G44575	NPQ4 (PSBS)	LHCII	0.23 (± 0.06)	0.38 (± 0.07)	0.36 (± 0.02)	0.33 (± 0.12)
AT1G34000	OHP2	aux	0.18 (± 0.01)	0.41 (± 0.13)	0.46 (± 0.17)	0.36 (± 0.10)
AT4G10340	CP26 (LHCB5)	PSII	0.16 (± 0.05)	0.25 (± 0.05)	0.22 (± 0.01)	0.20 (± 0.06)
AT5G64040	PSAN	PSI	0.16 (± 0.08)	0.40 (± 0.14)	0.50 (± 0.07)	0.42 (± 0.20)
AT5G02120	OHP1*	aux	0.14 (± 0.04)	0.26 (± 0.04)	0.29 (± 0.07)	0.23 (± 0.03)
AT1G29930	LHCB1.3	LHCII	0.11 (± 0.05)	0.08 (± 0.00)	0.18 (± 0.18)	0.27 (± 0.17)
AT3G47470	LHCA4	PSI	0.11 (± 0.02)	0.36 (± 0.39)	0.23 (± 0.11)	0.20 (± 0.01)
ATCG00280	CP43 (PSBC)*	PSII	0.07 (± 0.01)	0.15 (± 0.04)	0.17 (± 0.02)	0.13 (± 0.03)
AT3G54890	LHCA1	PSI	0.07 (± 0.01)	0.18 (± 0.14)	0.12 (± 0.02)	0.11 (± 0.02)
AT1G31330	PSAF	PSI	0.07 (± 0.04)	0.20 (± 0.17)	0.15 (± 0.05)	0.17 (± 0.06)
AT1G61520	LHCA3	PSI	0.07 (± 0.02)	0.20 (± 0.10)	0.15 (± 0.05)	0.11 (± 0.02)
AT1G52230	PSAH-2	PSI	0.06 (± 0.04)	0.38 (± 0.49)	0.22 (± 0.15)	0.15 (± 0.13)
AT2G46820	PSAP	PSI	0.06 (± 0.01)	0.12 (± 0.00)	0.18 (± 0.06)	0.12 (± 0.03)
AT1G79040	PSAR	PSI	0.05 (± 0.00)	0.07 (± 0.02)	0.08 (± 0.01)	0.08 (± 0.03)
AT3G08940	CP29-2 (LHCB4.2)	PSII	0.05 (± 0.01)	0.08 (± 0.02)	0.07 (± 0.01)	0.06 (± 0.01)
AT5G66570	PSBO-1	PSII	0.05 (± 0.02)	0.08 (± 0.03)	0.08 (± 0.03)	0.09 (± 0.06)
AT3G61470; AT5G28450	LHCA2; CAB family	PSI	0.04 (± 0.01)	0.10 (± 0.08)	0.08 (± 0.02)	0.06 (± 0.02)
ATCG00340	PSAB	PSI	0.04 (± 0.01)	0.16 (± 0.14)	0.12 (± 0.03)	0.09 (± 0.04)
AT1G15820	CP24 (LHCB6)	PSII	0.03 (± 0.01)	0.04 (± 0.01)	0.04 (± 0.01)	0.04 (± 0.01)
AT4G28750	PSAE-1	PSI	0.03 (± 0.02)	0.10 (± 0.06)	0.08 (± 0.01)	0.07 (± 0.04)
ATCG00350	PSAA	PSI	0.03 (± 0.00)	0.10 (± 0.07)	0.09 (± 0.02)	0.07 (± 0.01)
AT5G51545	LPA2	aux	0.03 (± 0.02)	0.04 (± 0.02)	0.07 (± 0.02)	0.06 (± 0.03)
AT5G01530	CP29-1 (LHCB4.1)	PSII	0.02 (± 0.01)	0.03 (± 0.00)	0.03 (± 0.01)	0.03 (± 0.01)
AT4G12800	PSAL	PSI	0.02 (± 0.01)	0.07 (± 0.07)	0.04 (± 0.02)	0.03 (± 0.02)
AT4G02770	PSAD-1	PSI	0.01 (± 0.00)	0.07 (± 0.07)	0.05 (± 0.02)	0.03 (± 0.02)
AT1G03600	PSB27	aux	0.01 (± 0.00)	0.03 (± 0.01)	0.03 (± 0.01)	0.04 (± 0.02)
ATCG00560	PSBL	PSII	0.01 (± 0.01)	0.02 (± 0.00)	0.02 (± 0.00)	0.02 (± 0.02)^a^
AT1G08380	PSAO	PSI	0.01^b^	0.07 (± 0.09)^a^	0.04^b^	0.01 (± 0.00)^a^
AT1G30380	PSAK	PSI	0.01 (± 0.00)	0.04 (± 0.05)	0.01 (± 0.01)	0.01 (± 0.00)

Thylakoid membranes were solubilized with digitonin, complexes separated using lpBN-PAGE and selected bands excised for LC–MS/MS analysis. Annotated PSI, PSII and LHC subunits as well as auxiliary (aux.) PS components are presented. The quantity of each protein was normalized to LHCB3, and the relative protein abundances between genotypes were compared using ANOVA/Brown-Forsythe. Post-hoc analysis of significantly different protein groups (ANOVA *p* < 0.05) was performed using Tukey-HSD. The average relative abundances of detected proteins (± standard deviation) are shown (*n* = 3; *N* = 12). The spectrum file was re-analyzed from Koskela et al. (2018) (www.plantcell.org, copyright American Society of Plant Biologists)

^*^ANOVA *p* < 0.05

^a^*n* = 2

^b^*n* = 1

For the 2D gel electrophoresis, lpBN gel strips were solubilized and run in a second dimension on 12% reducing SDS-PAGE supplemented with 6 M urea (Suorsa et al. 2015). Gels were stained with SYPRO™ total protein stain according to the manufacturer´s instructions.

### Statistical analyses for proteomics

iBAQ values can be used to calculate the relative protein abundances of different proteins within one complex (Schwanhäusser et al. 2011). However, they do not allow a direct comparison between different biological replicates or between genotypes, since the raw iBAQ values are not normalized between samples. To overcome this limitation, and to still retain the information of the relative protein abundance within one complex, we normalized the relative protein abundances within each complex to percentage values. Hence, the iBAQ values of all proteins in PSI-containing complexes were normalized to the intensity of the PSI reaction center protein PSAA, to compare the relative abundances of accessory PSI subunits, antenna proteins, and PSII subunits between the genotypes. Proteins detected in the LHCII trimer complexes were normalized to the LHCB3 subunit of PSII antenna, since this subunit is not present in the mobile L-LHCII antenna (Galka et al. 2012), and therefore, allows us to estimate whether there is a change in the distribution of different types of antenna trimers found in particular thylakoid domains (i.e. non-appressed thylakoids and whole thylakoids) between the genotypes. Only proteins whose abundance was more than 1.0% of PSAA/LHCB3 were included in the analysis, since very low abundance proteins were considered likely contaminants due to imperfect separation during lpBN gel electrophoresis. Additionally, only proteins with MS-score > 40 were considered reliably detected and included in the analysis. Normalized iBAQ values for each complex (digitonin-solubilized PSI complex and LHCII trimer, and DM-solubilized PSI/PSII dimer complex and LHCII trimer, Fig. 2) were used for statistical analyses with ANOVA in SPSS statistics software (IBM) and differences in the normalized relative abundances between genotypes were tested. If variances between genotypes were significantly different (Levene test *p* < 0.05), the robust Brown–Forsythe test was used instead. If a protein was detected in only one replicate for a genotype, that genotype was omitted from statistical analysis for that protein. Missing values (i.e. cases where protein was not detected in a replicate) were set as ‘system missing’. Because peptide/protein abundances were found to be significantly different (ANOVA or Brown–Forsythe *p* < 0.05) only in cases where variances between groups were homogenous, post hoc analysis was performed using Tukey-HSD. *p *values for statistical analyses are shown in Supplemental Tables 3–7.

## Results

### *stn7* and *nsi* mutants under fluctuating light conditions

**Fig. 1:**
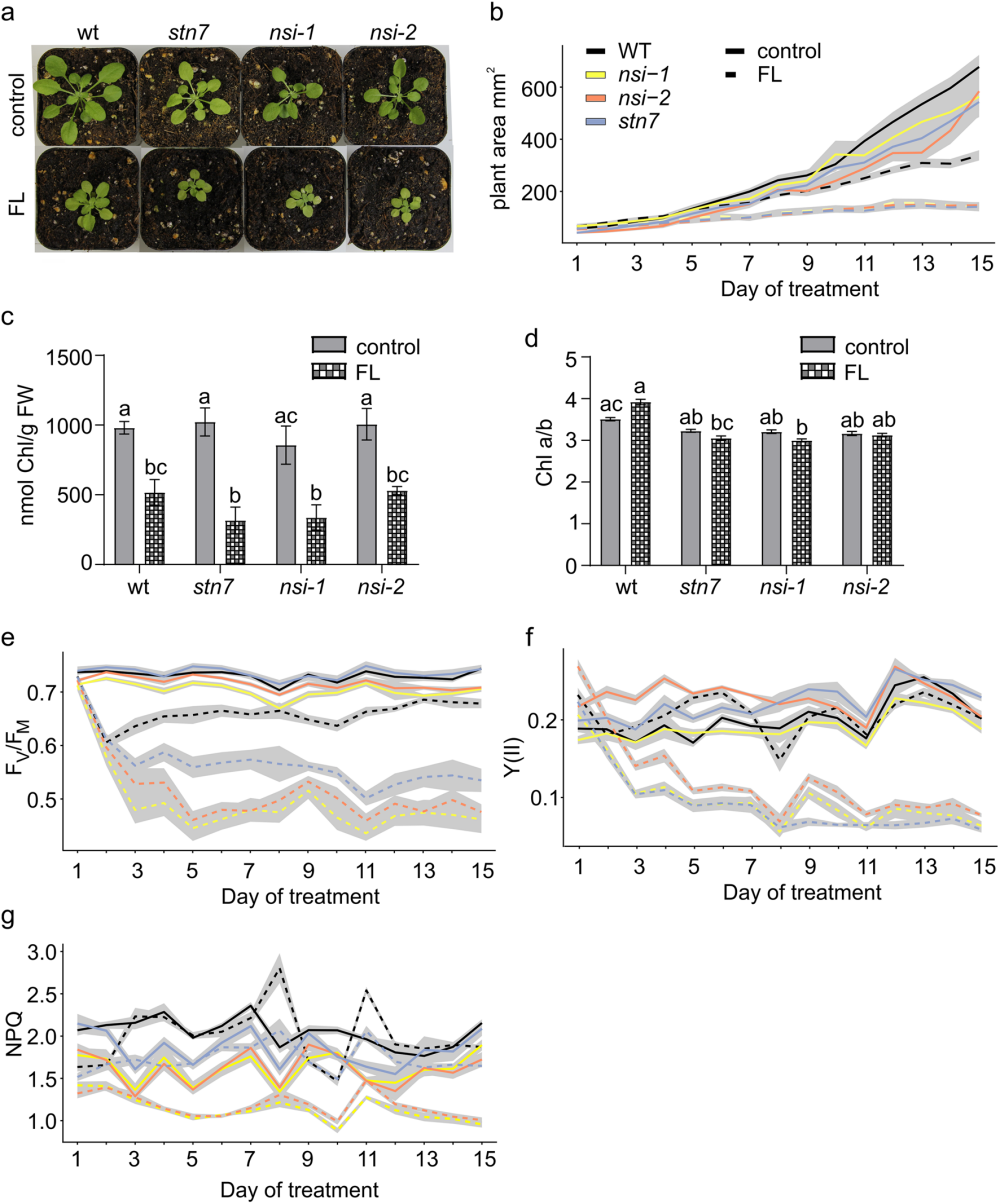
Growth and photosynthetic properties of *Arabidopsis thaliana* wild type (wt), *stn7* and *nsi* plants grown under standard and fluctuating light conditions. **a** Visual phenotype, **b** area, **c** chlorophyll (Chl) a and b content, **d** Chl a/b ratio, **e** maximum quantum efficiency of PSII photochemistry (monitored as *F*_V_/*F*_M_), **f** steady-state PSII yield (Y(II)), **g** non-photochemical quenching (NPQ/4) of the plants grown under standard conditions for 4 weeks (control) or for 2 weeks under standard conditions followed by 2 weeks growth under fluctuating light (FL; 4 min of 90 μmol photons m^−2^ s^−1^ and 1 min of 900 μmol photons m^−2^ s^−1^). **a**, **c**, **d** represent values from mature plants and **b**, **e**, **f**, **g** show results from daily measurements during the 2 weeks FL treatment. Black line in **b**, **e**, **f** and **g** denotes wt, blue *stn7*, yellow *nsi-1* and orange *nsi-2*. Solid lines represent plants grown under standard light conditions and dotted lines FL treatment. **f**, **g** shows average of last two data points on the induction curve. Shaded region in **b**, **e**, **f**, **g** represents + / − 1 standard error. Six plants were used per genotype and per treatment (4)

The *stn7* knock-out plants are deficient in the short-term regulation of the photosynthetic light reactions via LHCII phosphorylation (Depège et al. 2003; Bellafiore et al. 2005). Interestingly, the *stn7* mutant does not show growth defects under constant light conditions, but exposure to fluctuating light causes a dramatic retardation in growth (Bellafiore et al. 2005; Tikkanen et al. 2010). The *nsi* loss-of-function lines are not compromised in the phosphorylation of thylakoid proteins, but they are incapable of performing state transitions (Koskela et al. 2018). Nevertheless, the phenotype of the *nsi* plants did not markedly differ from wt controls under standard growth conditions (Koskela et al. 2018). To find out whether the retarded growth of *stn7* under fluctuating light is due to defects in state transitions alone, 2-week old wt, *nsi-1, nsi-2,*and *stn7* plants were treated for 2 weeks with fluctuating light (cycles of 4 min 90 µmol photons m^−2^ s^−1^ followed by a 1 min 900 µmol photons m^−2^ s^−1^). Indeed, *stn7* as well as both *nsi* lines (*nsi-1* and *nsi-2*) showed significantly reduced growth under fluctuating light conditions (Fig. 1a, b). The fluctuating light treatment also decreased the chlorophyll content of all plant lines (Fig. 1c). In wt, fluctuating light increased the chlorophyll a/b ratio, while in all mutant lines the chlorophyll a/b ratio slightly decreased (Fig. 1d).

To gain insights into the photosynthetic properties of the *nsi* and *stn7* plants, maximum PSII quantum yield (*F*_V_/*F*_M_) of the plants grown under standard growth conditions or treated with fluctuating light (cycles of 4 min 90 µmol photons m^−2^ s^−1^ followed by a 1 min 900 µmol photons m^−2^ s^−1^) were determined using an IMAGING-PAM fluorometer. *F*_V_/*F*_M_ in wt plants decreased sharply within the two first days of fluctuating light treatment as compared to the plants grown under standard conditions. However, during the 2-week treatment, the PSII capacity recovered almost to the same level as detected under standard growth conditions (Fig. 1e). In contrast, *F*_V_/*F*_M_ in the three loss-of-function mutant lines revealed a uniform and drastic decrease, which did not recover throughout the 2-week fluctuating light treatment (Fig. 1e). In line with maximum PSII efficiency (F_V_/F_M_), fluctuating light did not affect the PSII yield (YII) of wt plants (Fig. 1f). In all mutant lines, however, the PSII yield was drastically decreased by the fluctuating light treatment (Fig. 1f). In both *stn7* and wt, the level of steady-state NPQ was not dramatically affected by fluctuating light treatment, as compared to constant light, even if in *stn7* NPQ was consistently lower than in wt (Fig. 1g). In both *nsi* lines, NPQ levels were even lower than in *stn7* even under standard growth conditions. Intriguingly, fluctuating light treatment of the plants resulted in a drastic decrease in NPQ only in the *nsi* mutants (Fig. 1g). Moreover, fluctuating light treatment resulted in an increase of quantum yield of non-regulated energy dissipation Y(NO), expressed as the average of the last two data points on the induction curve on day 15 (*n* = 6), especially in the *nsi* mutant plants: for *nsi-1*, Y(NO) increased from 0.31 ± 0.02 under standard light conditions to 0.55 ± 0.02 under fluctuating light, and for *nsi-2* the increase was from 0.31 ± 0.02 to 0.54 ± 0.02. In *stn7*, the increase was smaller (from 0.27 ± 0.02 to 0.41 ± 0.01) than in *nsi*. In contrast to the mutants, Y(NO) in wt remained constant (0.28 ± 0.02) under standard conditions and under fluctuating light (0.29 ± 0.02).

### Composition of thylakoid protein complexes

**Fig. 2:**
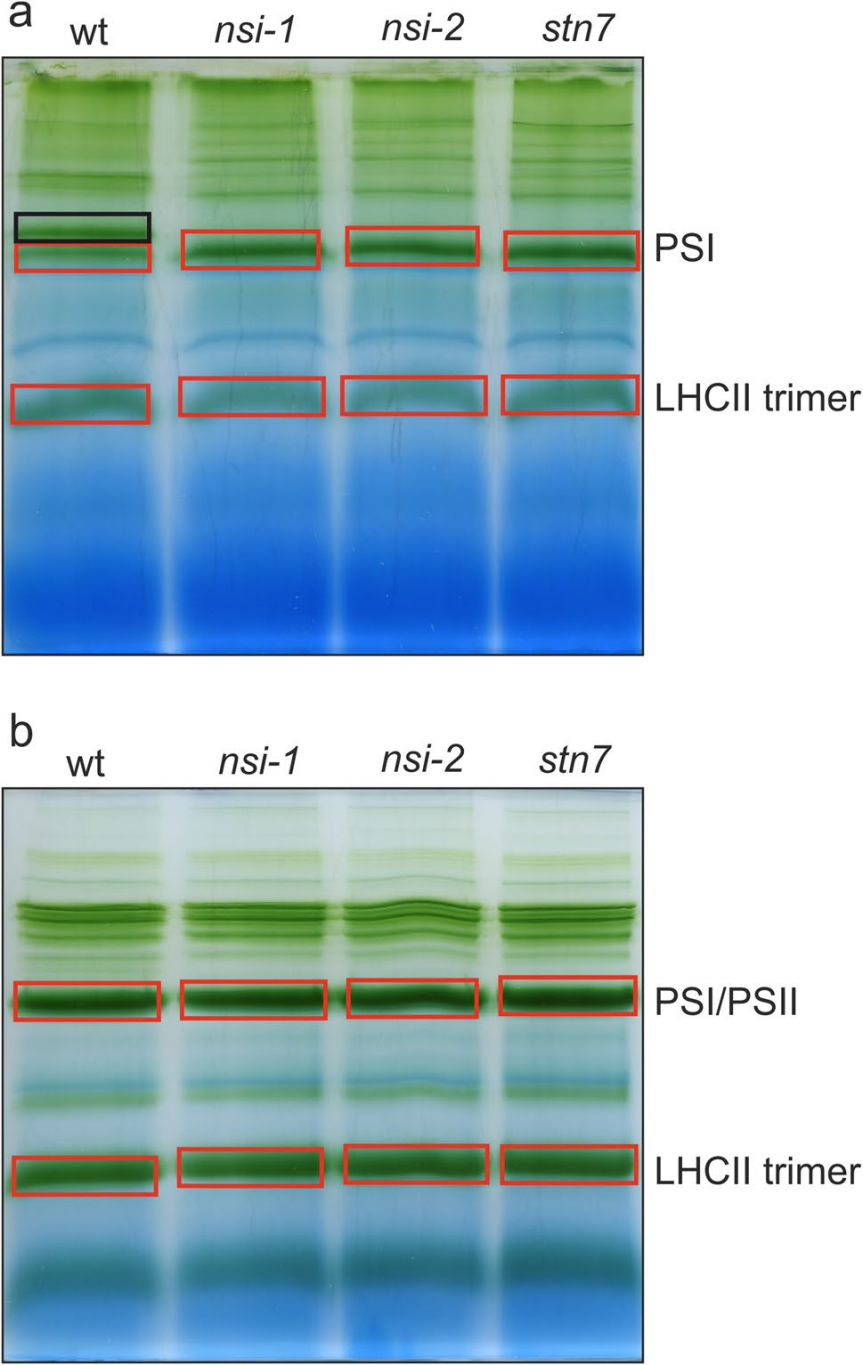
Thylakoid protein complexes of wt, *nsi-1*, *nsi-2,* and *stn7* plants. Plants were grown under standard conditions (100 µmol photons m^−2^ s^−1^, 8 h/16 h light/dark), thylakoids solubilized either with **a** 1% (w/v) digitonin (solubilization of grana margins and stroma lamellae) or **b** 1% (w/v) dodecyl maltoside (solubilization of the entire thylakoid network), and protein complexes separated using lpBN gel electrophoresis. 50 µg of chlorophyll was loaded in (**a**) and 30 µg in (**b**). Slices indicated by red boxes were cut from the gels and subjected to LC–MS/MS. The black box indicates the PSI–LHCII complex (state transition complex) present in the grana margins or stroma lamellae of wt plants

**Fig. 3:**
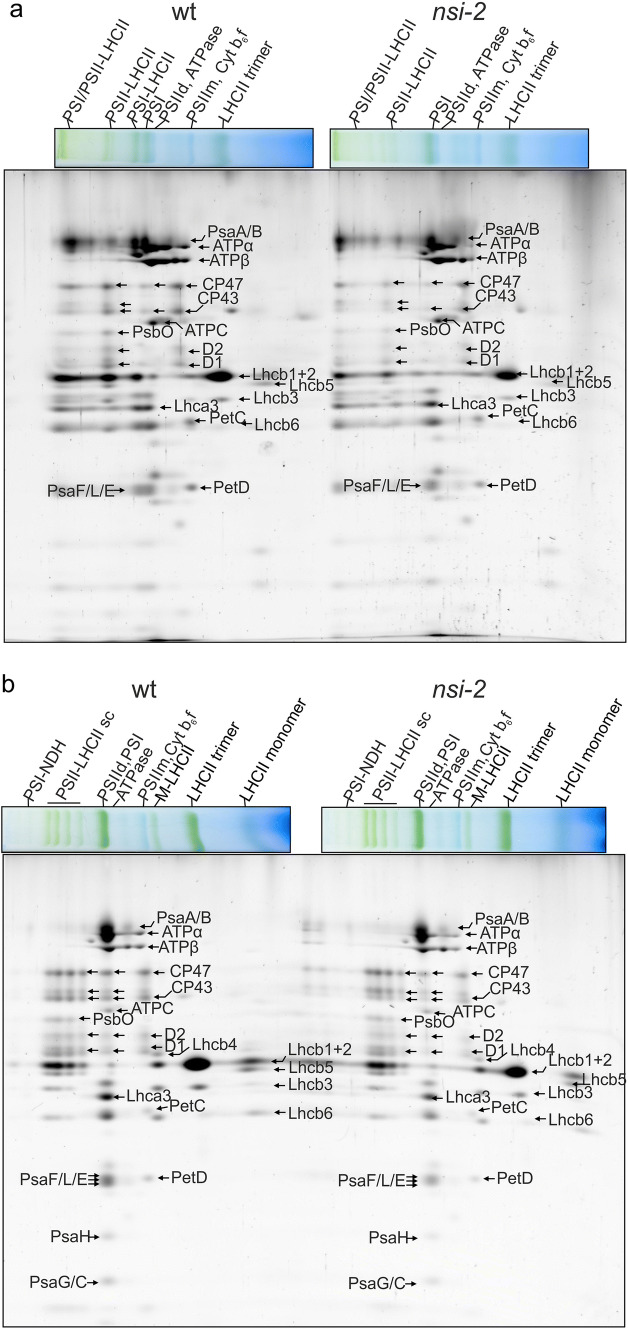
2D-Blue Native gels of wt and *nsi-*2 thylakoid protein complexes. Thylakoid samples from growth light (100 µmol photons m^−2^ s^−1^) adapted plants were solubilized either with **a** 1% (w/v) digitonin (5 µg of chlorophyll) or **b** 1% (w/v) dodecyl maltoside (3.5 μg of chlorophyll) prior to lpBN gel electrophoresis. lpBN gel electrophoresis was followed by separation of protein complexes in the second dimension on 12% reducing SDS-PAGE supplemented with 6 M urea and SYPRO staining. The proteins were identified based on Aro et al. 2005 and Suorsa et al. 2015. sc, supercomplex; PSIId, PSII dimer; PSIIm, PSII monomer; Cytb_6_f, cytochromes b_6_f

To define the structural differences of the main players of state transitions, i.e. PSI and LHCII, between wt, *stn7* and *nsi* mutants, the plants were grown under standard conditions for 5 weeks, and thylakoids were isolated in the middle of the light period, where wild type plants are in state 2 (Suorsa et al. 2015). Thereafter, thylakoids were solubilized with commonly used detergents digitonin or β-DM (hereafter DM). Digitonin has been used to study weak protein–protein interactions in the non-appressed regions of thylakoids, while DM solubilizes the entire thylakoid membrane, but is unable to maintain labile interactions between the protein complexes (Järvi et al. 2011). Indeed, the chlorophyll a/b ratio of the digitonin-solubilized wt thylakoid fraction was 4.34 ± 0.244 and that of the DM-solubilized thylakoids 3.21 ± 0.033 (*n* = 3). As the chlorophyll a/b ratio of total thylakoids was 3.20 ± 0.022, it is clear that DM solubilizes the entire thylakoid membrane, while digitonin solubilizes less Chl b-containing grana membranes (i.e. non-appressed thylakoid fraction). These values are in accordance with the previously published results (e.g. Kyle et al. 1983; Rantala et al. 2017; Koochak et al. 2019; Trotta et al. 2019) and also with the results obtained by mechanical fractionation (e.g. Danielsson et al. 2006; Suorsa et al. 2014). Thereafter, solubilized thylakoid protein complexes were separated using lpBN gel electrophoresis, and selected protein complexes were analyzed by LC–MS/MS (Fig. 2). As previously shown, the overall accumulation of the thylakoid protein complexes was similar in all plant lines, and no visual differences could be detected when thylakoids were solubilized with DM (Fig. 2b; Tikkanen et al. 2008; Koskela et al. 2018). Nevertheless, the well-characterized state transition complex composed of PSI, LHCI and LHCII was missing in *stn7* and *nsi* mutants, when thylakoids were solubilized with digitonin, and also the megacomplex composition of the mutants differed from that of the wt (Fig. 2a; Pesaresi et al. 2009; Suorsa et al 2015; Koskela et al. 2018). We also took a closer look at the thylakoid protein complexes of *nsi* by running a 2D-BN gel, but apart from the lack of PSI–LHCII complex, no major differences in the subunit composition of *nsi-1* or *nsi-2* thylakoid complexes could be detected as compared to wt (Fig. S1, Fig. 3).

#### PSI/PSII band

The PSI/PSII band of the non-appressed thylakoids, obtained by digitonin solubilization and lpBN gel electrophoresis (defined in Fig. 2a), was analyzed by LC–MS/MS. This band has previously shown to contain mainly PSI subunits (Suorsa et al. 2015), which was also the major constituent of the band in our iBAQ analysis (Table 1). Comparison of wt and *stn7* and *nsi* mutant lines showed equal accumulation of the PSI core subunit PSAB, PSI proteins facing the lumen (PSAN and PSAF) as well as the PSI proteins forming the stromal ridge (PSAC, PSAD and PSAE) responsible for ferredoxin recruitment (Table 1, Supplemental Table S1). Neither the relative abundance of the proteins involved in the binding of LHCII to PSI (PSAH, PSAL and PSAO) (Lunde et al. 2000; Schöttler et al. 2011; Pan et al. 2018) nor the minor PSI proteins (PSAG, PSAK, PSAP) differed between the mutant lines as compared to wt. Moreover, no differences were detected in the accumulation of LHCA1–4 proteins (Table 1). Small amounts of PSII core dimers have been shown to co-migrate in this band together with PSI complexes (Suorsa et al. 2015; Rantala et al. 2017). Our analysis confirmed that PSII proteins were less abundant (only up to 25%) in this band as compared to PSAA. PSII core proteins D1 and D2, inner antenna proteins PSBB and PSBC, as well as the minor PSII protein PSBL accumulated in equal quantities in all plant lines (Table 1). It should be noted, however, that the low abundance of PSII proteins does not allow reliable estimation of PSI/PSII ratio within this band and would not be relevant since the majority of PSII complexes are left insolubilized during digitonin solubilization and are thus not involved in the analysis. Oxygen-evolving complex proteins PSBO-1, PSBO-2 and PSBR were also represented within the samples. Interestingly, the amount of PSBO-2 in the PSII dimer migrating in the PSI/PSII band was decreased to around 60% in the *nsi* mutants as compared to wt (*p* =  0.004 and 0.011 for *nsi-2* and *nsi-1*, respectively). In *stn7* the accumulation of PSBO-2 was also slightly decreased compared to wt (around 85% of wt level), but the difference was not significant (*p* = 0.374), while a significant difference remained between *nsi-2* and *stn7* (*p* = 0.043). Similarly, although less pronounced, *nsi-1* had less PSBO-2 than *stn7* (*p* = 0.114). The PSBO-1 isoform also showed lower accumulation in the *nsi* mutants as compared to wt, although with less significance compared to PSBO-2 (*p *values = 0.054 and 0.075 for *nsi-1* and *nsi-2*, respectively). Although mainly photosystem core complexes are known to migrate in this band, also several peptides representing different isoforms of LHCB1 (LHCB1.1/1.2, LHCB1.5), LHCB2 (LHCB2.1/2/4) and LHCB4 (LHCB4.1, LHCB4.2, LHCB4.3) as well as LHCB3, LHCB5 and LHCB6 proteins were detected in all plant lines. Significantly less peptides, around 40% of wt level, representing LHCB1.1 and LHCB1.2 were observed in the PSI/PSII band of digitonin-solubilized *stn7* thylakoids as compared to wt (Table 1) (*p* = 0.019). In *nsi* mutants, the amount of LHCB1.1/1.2 in the band was also reduced to 60% in *nsi-1* (*p* = 0.142) and 50% in *nsi-2* (*p* = 0.019) when compared with wt, while there was no significant difference between *nsi* and *stn7* mutants. However, as the protein complex pattern in wt is very different from that of the mutants (which lack the PSI–LHCII complex), direct comparison of the LHCII protein abundance to PSI is not possible.

The PSI/PSII band was also analyzed from thylakoid samples solubilized with DM (band defined in Fig. 2b), and it was shown to contain a similar complement of proteins as did the PSI/PSII band from thylakoids solubilized with digitonin (Table 2). The most abundant PSI proteins (normalized to PSAA) were PSAD, PSAE, PSAC, and PSAG (Table 2). In addition, many other PSI subunits as well as LHCA1–4 proteins were detected. Since DM-solubilized thylakoids contain also the appressed grana thylakoids, which host the majority of the PSII complexes, the PSII proteins were more abundant in the PSI/PSII band of DM-solubilized samples as compared to digitonin-solubilized samples (Table 2), reaching up to half of the PSAA amount. This result is in line with earlier studies showing enrichment of PSI subunits in digitonin-solubilized thylakoids (Rantala et al. 2017; Koochak et al. 2019; Trotta et al. 2019). Interestingly, the CP43/PSAA ratio in the PSI/PSII band of the DM-solubilized thylakoids was 30% lower in *nsi* as compared to *stn7* (*p* = 0.023 and 0.085 for *nsi-2* and *nsi-1*, respectively). In fact, a similar trend was observed for all PSII core subunit (D1, D2, CP43 and CP47): their amount was slightly reduced in *nsi* as compared to *stn7* and wt, although the differences were not statistically significant apart from the above-mentioned CP43. Moreover, 2D gel electrophoresis analysis revealed that the amount of phospho-CP43 (upper band) seemed higher and the amount of non-phosphorylated CP43 (lower band) lower in *nsi-2* as compared to wt (Fig. 3).

Similar to the non-appressed thylakoids, analysis of the PSI/PSII band of DM-solubilized thylakoids showed that *nsi* mutants contained less PSBO-2 than wt and *stn7*. However, in DM-solubilized thylakoids, the difference between *nsi* and wt was less pronounced (PSBO-2 level of *nsi*s around 85% of wt) and statistically non-significant, whereas the amount in *stn7* was increased compared to wt (*p* = 0.095). Overall, the only statistically significant difference in PSBO-2 amount in whole thylakoids was between both *nsi* mutants and *stn7* (*p* = 0.044 and 0.048 for *nsi-2* and *nsi-1*, respectively), with *nsi-1* and *nsi-2* having around 40% less PSBO-2 in this complex.

#### LHCII trimers

During lpBN gel electrophoresis, large amount of LHCII trimers (Fig. 2) disconnect from the photosystems due to solubilization (Tikkanen et al. 2008, 2012). Previously, it has been shown that LHCB1 and LHCB2 are the most abundant proteins in the disconnected “free” LHCII trimers (Galka et al. 2012). Accordingly, Table 3 shows that LHCII trimers in digitonin-solubilized thylakoids possessed high quantities of LHCB2.2 and LHCB1.5 as normalized to LHCB3, which, in line with previous findings (Galka et al. 2012; Rantala et al. 2017), was ten times less abundant than LHCB2.2. In addition to LHCB2.2, LHCB1.5 and LHCB3, the trimers contained LHCB1.4 and traces of LHCB1.3, LHCB5 and LHCB4.1–4.3. In addition, a number of PSII and PSI proteins as well as LHCA1–LHCA4 were detected, and the amount of CP43 was higher in both *nsi* mutants as compared to wt and *stn7*. Additionally, in all plant lines, the LHCII trimer complex contained equal amounts of some regulatory proteins involved in PSII biogenesis and repair (i.e. OHP2, PSB27), and the OHP1 protein showed significantly higher accumulation in both *nsi* mutant lines as compared to wt and *stn7* (Table 3). It should be noted, however, that at least part of the detected proteins in each band may be contaminants or protein aggregates co-migrating with the true components of the LHCII trimers. Moreover, our previous analysis has evidenced acetylation of the LHCB2.2 protein in the LHCII trimers of both *stn7* and the wild type in two to three replicates, while it was not detected at all in *nsi-1* and only once in *nsi-2* (Koskela et al. 2018).

In contrast to digitonin, DM effectively solubilizes the entire thylakoid membrane, including grana stacks and it has been shown that with DM, larger amount of the LHCII trimers disconnect from the photosystems when compared to digitonin (Rantala et al. 2017). MS analysis of the LHCII trimer from the DM samples (defined in Fig. 2b) demonstrated that LHCB2.2, LHCB1.4 and LHCB1.5 were the most abundant LHCB proteins (also when grana stacks were included), and that the relative portion of LHCB1.4 (compared to LHCB3) in LHCII trimers was increased tenfold as compared to digitonin-solubilized samples (Table 4). These complexes also contained significant amounts of LHCB1.1/2, which was absent in the LHCII trimers of the digitonin-solubilized thylakoids. LHCB4.1, LHCB4.2, LHCB5, and LHCB6 were much more abundant in whole thylakoids than in the non-appressed thylakoids, in agreement with their preferential localization of PSII complexes enriched in grana cores. In contrast, LHCB1.3 was solely detected in digitonin-solubilized thylakoids, suggesting a preferential localization in stroma thylakoids. Additionally, LIL3.1 and LIL3.2 proteins, which are required for chlorophyll and tocopherol biosynthesis (Tanaka et al. 2010; Lohscheider et al. 2015), were detected in the LHCII trimers in whole thylakoids. Some differences were also found in the accumulation of PSI and PSII proteins in the LHC trimers between the DM- and digitonin-solubilized thylakoids. PSAH-1, PSAO-2, PSBQ-1 and PSBR were only detected in whole thylakoids, while PSAP, PSAR and PSBL were detected only in grana margins and stroma lamellae. In the DM LHCII trimer, PSBQ-2 was more abundant in *nsi-2* than in wt (Table 4).

**Table 4:** Quantitative proteome data analysis according to iBAQ values of the proteins identified within the LHCII trimer bands excised from lpBN-PAGE gels

Proteins/protein groups		Subunit of	Genotype			
Locus/loci	Name(s)		wt	*nsi-1*	*nsi-2*	*stn7*
AT2G05070	LHCB2.2	LHCII	9.86 (± 1.53)	8.50 (± 2.91)	10.96 (± 1.11)	13.47 (± 4.69)
AT2G34430	LHCB1.4	LHCII	5.28 (± 1.20)	4.78 (± 2.36)	3.06 (± 0.00)	2.25 (± 1.00)
AT2G34420	LHCB1.5	LHCII	2.22 (± 0.00)	2.17 (± 1.04)	2.77 (± 0.00)	4.40 (± 2.60)
AT1G29920; AT1G29910	LHCB1.1;LHCB1.2	LHCII	1.20 (± 0.00)	1.31 (± 0.00)	1.02 (± 0.00)	1.36 (± 0.00)
AT5G54270	LHCB3	LHCII	1.00	1.00	1.00	1.00
AT4G10340	CP26 (LHCB5)	PSII	0.27 (± 0.00)	0.14 (± 0.00)	0.21 (± 0.00)	0.19 (± 0.00)
AT3G08940	CP29.2 (LHCB4.2)	PSII	0.18 (± 0.00)	0.17 (± 0.00)	0.16 (± 0.00)	0.62 (± 0.00)
ATCG00680	CP47 (PSBB)	PSII	0.16 (± 0.00)	0.15 (± 0.00)	0.15 (± 0.00)	0.51 (± 0.00)
ATCG00270	D2 (PSBD)	PSII	0.14 (± 0.00)	0.13 (± 0.00)	0.16 (± 0.00)	0.17 (± 0.00)
AT3G16140	PSAH-1	PSI	0.11 (± 0.00)	0.09 (± 0.00)	0.21 (± 0.00)	0.59 (± 0.00)
ATCG00020	D1 (PSBA)	PSII	0.10 (± 0.00)	0.09 (± 0.00)	0.12 (± 0.00)	0.16 (± 0.00)
AT5G66570	PSBO-1	PSII	0.08 (± 0.00)	0.08 (± 0.00)	0.10 (± 0.00)	0.10 (± 0.00)
AT3G47470	LHCA4	PSI	0.08 (± 0.00)	0.06 (± 0.00)	0.07 (± 0.00)	0.17 (± 0.00)
AT1G44575	NPQ4 (PSBS)	aux	0.07 (± 0.00)	0.07 (± 0.00)	0.09 (± 0.00)	0.06 (± 0.00)
AT5G01530	CP29.1 (LHCB4.1)	PSII	0.07 (± 0.00)	0.04 (± 0.00)	0.05 (± 0.00)	0.11 (± 0.00)
AT1G15820	CP24 (LHCB6)	PSII	0.06 (± 0.00)	0.05 (± 0.00)	0.05 (± 0.00)	0.09 (± 0.00)
AT1G31330	PSAF	PSI	0.06 (± 0.00)	0.05 (± 0.00)	0.05 (± 0.00)	0.05 (± 0.00)
ATCG00340	PSAB	PSI	0.05 (± 0.00)	0.04 (± 0.00)	0.06 (± 0.00)	0.14 (± 0.00)
AT5G47110	LIL3.2	aux	0.05 (± 0.00)	0.05 (± 0.00)	0.07 (± 0.00)	0.06 (± 0.00)
AT4G17600	LIL3.1	aux	0.05 (± 0.00)	0.04 (± 0.00)	0.06 (± 0.00)	0.05 (± 0.00)
AT1G61520	LHCA3	PSI	0.05 (± 0.00)	0.05 (± 0.00)	0.05 (± 0.00)	0.08 (± 0.00)
AT1G34000	OHP2	aux	0.05 (± 0.00)	0.06 (± 0.00)	0.10 (± 0.00)	0.04 (± 0.00)
ATCG00280	CP43 (PSBC)	PSII	0.04 (± 0.00)	0.04 (± 0.00)	0.04 (± 0.00)	0.10 (± 0.00)
AT3G54890	LHCA1	PSI	0.04 (± 0.00)	0.03 (± 0.00)	0.03 (± 0.00)	0.03 (± 0.00)
AT4G28750	PSAE-1	PSI	0.03 (± 0.00)	0.02 (± 0.00)	0.04 (± 0.00)	0.04 (± 0.00)
AT3G61470	LHCA2	PSI	0.03 (± 0.00)	0.02 (± 0.00)	0.02 (± 0.00)	0.05 (± 0.00)
AT5G02120	OHP1	aux	0.03 (± 0.00)	0.04 (± 0.00)	0.05 (± 0.00)	0.03 (± 0.00)
AT4G12800	PSAL	PSI	0.02 (± 0.00)	0.02 (± 0.00)	0.03 (± 0.00)	0.08 (± 0.00)
AT5G64040	PSAN	PSI	0.02 (± 0.00)	0.02 (± 0.00)	0.04 (± 0.00)	0.02 (± 0.00)
ATCG00350	PSAA	PSI	0.02 (± 0.00)	0.02 (± 0.00)	0.03 (± 0.00)	0.06 (± 0.00)
AT1G30380	PSAK	PSI	0.02 (± 0.00)	0.02 (± 0.00)	0.03 (± 0.00)	0.12 (± 0.00)
AT4G02770	PSAD-1	PSI	0.02 (± 0.00)	0.01 (± 0.00)	0.02 (± 0.00)	0.04 (± 0.00)
AT1G79040	PSBR	PSII	0.02 (± 0.00)	0.02 (± 0.00)	0.01 (± 0.00)	0.01 (± 0.00)
AT1G52230	PSAH-2	PSI	0.01 (± 0.00)	0.01 (± 0.00)	0.01 (± 0.00)	0.03 (± 0.00)
AT1G08380	PSAO	PSI	0.01^a^	0.01^a^	0.02^a^	0.04^a^
AT3G50820	PSAO-2	PSI	0.01 (± 0.00)	0.01 (± 0.00)	0.01 (± 0.00)	0.01 (± 0.00)
AT4G21280	PSBQ-1	PSII	0.01 (± 0.00)	0.01 (± 0.00)	0.01 (± 0.00)	0.01 (± 0.00)

Thylakoid membranes were solubilized with dodecyl maltoside, complexes separated using lpBN-PAGE and selected bands excised for LC–MS/MS analysis. Annotated PSI, PSII and LHC subunits as well as auxiliary PS (aux.) components are presented. The quantity of each protein was normalized to LHCB3, and the relative protein abundances between genotypes were compared using ANOVA/Brown-Forsythe. Post-hoc analysis of significantly different protein groups (ANOVA *p* < 0.05) was performed using Tukey-HSD. The average relative abundances of detected proteins (± standard deviation) are shown (*n* = 3; *N* = 12)

^a^*n* = 1

We also analyzed the lysine acetylation status of the proteins present in the PSI/PSII band and LHCII trimers (Tables S1 and S2). It should be noted, however, that the low concentration of proteins eluted from the lpBN gel bands prohibited the usual antibody-based enrichment of acetylated peptides. This limitation resulted in markedly lower number of detected acetylated peptides as compared to total acetylome analysis (Koskela et al. 2018). Nevertheless, several PSI, PSII, LHCI and LHCII subunits, including PSAA/PSAB, PSAD, PSAH, PSBO2, LHCA1, LHCB1 and LHCB2, were found to be lysine-acetylated (Tables S1 and S2).

## Discussion

Plants are well equipped for acclimation to major fluctuations they experience in their natural environments. For instance, changes in ambient illumination require safe quenching and/or rapid adjustments in the distribution of absorbed energy between the photosystems. The LHCII complex is in the center of these adjustments, as it may either absorb or quench light energy, and regulate energy distribution by associating with either PSI or PSII (Rochaix 2014). The *stn7* mutants, lacking LHCII phosphorylation, show retarded growth when exposed to fluctuating light conditions, which indicates an important role for LHCII phosphorylation and state transitions in response to environmental changes (Bellafiore et al. 2005; Tikkanen et al. 2010). Our results show delayed growth and reduced accumulation of chlorophyll in the *stn7* and the two *nsi* mutants under fluctuating light (Fig. 1a-c), supporting the view that state transitions are indeed required to adapt to fluctuating light conditions. In line with the reduced growth, and in contrast to those of wt, the PSII capacity (*F*_V_/*F*_M_) and yield (Y(II)) of all mutant lines showed a substantial decrease with fluctuating light treatment (Fig. 1e,f). The drastic decrease of NPQ detected in both *nsi* lines but not in *stn7* under fluctuating light as compared to standard light conditions indicates that the capacity of the *nsi* plants to quench absorbed light energy is severely disturbed (Fig. 1g). In our previous study, we have shown that under high light illumination (> 200 µmol photons m^−2^ s^−1^) the *nsi* plants grown under standard conditions exhibit higher steady-state NPQ than wt (Koskela et al. 2018). Future studies are needed to pinpoint the reason behind the differential NPQ capacity of *nsi* plants: is it a consequence of decreased formation of lumenal ΔpH, differential accumulation of PSBS or xanthophylls at the thylakoids (Derks et al. 2015), or decreased Lys acetylation status of some LHC proteins detected in *nsi* mutants (Koskela et al. 2018)?

Application of DM and digitonin solubilization followed by lpBN gel electrophoresis and LC–MS/MS analysis allowed us to conclude that PSII complexes are indeed enriched in grana stacks as compared to grana margins and stroma thylakoids, which is in line with the dogma of lateral heterogeneity of thylakoids (Albertsson 2001; Tikkanen et al. 2012; Pribil et al. 2014). The most abundant LHCII proteins in the free LHCII trimers present in the non-appressed thylakoids were LHCB2.2, LHCB1.5, LHCB3 and LHCB1.4 (Table 3). It has been shown that the L-LHCII trimers are enriched with LHCB1.4, LHCB1.5 and LHCB2, while LHCB3 (and LHCB1.4) proteins are abundant in M-LHCII, which contain very little LHCB1.5 and LHCB2 (Galka et al. 2012). S-LHCII trimers, in turn, are enriched with LHCB1.1–3 (Galka et al. 2012). As only relatively low amount of LHCB1.1–3 protein were detected in the LHCII trimer band, it seems conceivable that the LHCII trimer mainly represents the L-LHCII trimers accompanied with some M-LHCII trimer (Table 3). The absence (or low quantity) of LHCB1.1–3 in the LHCII trimer of grana margins and stroma lamellae (Table 3), together with the fact that LHCB1.1–2 and LHCB2 proteins were more abundant than LHCB1.4 and LHCB1.5 in the PSI/PSII complex (Table 1), suggests that the S-LHCII trimers have remained attached to PSII, which is in line with previous findings (Boekema et al. 1999; Kouril et al. 2012; Rantala et al. 2017). M-LHCII trimers appeared to be more evenly distributed between the PSII-bound pool and LHCII trimer pool.

Under standard growth conditions, the L-LHCII trimers in the grana margins (and stroma lamellae) of wt plants are associated with PSI forming the state transition complex, while such complex formation and consequent energy transfer is not detected in *stn7* and *nsi* (Figs. 2 and 3). Intriguingly, no differences could be detected in the composition and abundance of LHCII proteins in the LHCII trimer band between the different plant lines (Tables 3 and 4). This result may suggest that the L-LHCII trimers in *stn7* and *nsi* remain tightly attached to the PSII–LHCII (even upon strong phosphorylation, as in *nsi*), because if the PSII–LHCII interaction was weakened, higher accumulation of LHCB2 and LHCB1.4–5 in the LHCII trimers in *stn7* and *nsi* than in wt would be expected. However, as the pool of L-LHCII capable of associating with PSI is very small (Järvi et al. 2011; Grieco et al. 2015), this effect may be masked by the high total quantity of LHCII. Moreover, it has been shown that the total content of LHCB1 is markedly downregulated in *stn7*, with a concomitant increase in the amount of LHCB2 (Tikkanen et al. 2006). The decrease in the LHCB1 amount in *stn7* was evident in the PSI/PSII complex of non-appressed thylakoid membranes, but we did not detect an increase in LHCB2 content (Table 2). Moreover, as the composition of LHCII proteins in the LHCII trimer band did not differ between the lines, it is possible that the shift in LHCB1/LHCB2 ratio concerns mainly the LHCII trimers that are forming the high molecular weight PSII–LHCII supercomplexes and PSI–PSII–LHCII megacomplex (Suorsa et al. 2015; Rantala et al. 2017), which were not investigated in this study.

The OHP2 protein was present in the PSI/PSII complexes of grana margins and stroma lamellae, but not in those of grana stacks (Supplemental Tables S1 and S2). This is in agreement with the recently resolved role of OHP1 (and OHP2) in the early stages of PSII de novo assembly and repair under high light conditions (Myouga et al. 2018; Li et al. 2019). OHP1 and OHP2 proteins belong to the light harvesting-like protein family, which contain a chlorophyll-binding domain and one transmembrane helix (Adamska et al. 1999; Rochaix and Bassi 2019). Intriguingly, OHP1 protein amount was significantly higher in the LHCII trimers of non-appressed thylakoids in both *nsi* mutants as compared to wt and *stn7* (Table 1). Further studies are needed to clarify the role of OHP1 in the *nsi* plants. Another marked difference between the plant lines was the decreased accumulation of PSBO proteins in the PSI/PSII complexes of non-appressed thylakoids in *nsi* as compared to wt and *stn7* (Table 1). *PSBO-1* and *PSBO-2* genes encode the extrinsic PSBO proteins in the PSII oxygen-evolving complex (De Las Rivas et al. 2004), and play an important role in stabilization of the Mn_4_Ca-cluster (Miyao and Murata 1984). PSBO-1 is the major isoform in Arabidopsis required for the optimal lateral migration of PSII complexes between the grana and stroma lamellae in response to high light illumination (Allahverdiyeva et al. 2009). As the total acetylome analysis showed decreased acetylation of another OEC protein, i.e. PSBP in the *nsi* mutants as compared to wt (Koskela et al. 2018), OEC might play a role in the dynamic formation of protein megacomplexes at the thylakoid membrane in response to changes in ambient illumination. Indeed, PSBO is required for binding of PSBP to PSII (Kavelaki and Ghanotakis 1991), while PSBQ stabilizes the association between PSBP and PSBO (Kakiuchi et al. 2012; Bricker et al. 2012; Allahverdiyeva et al. 2013). It has also been shown that deficiency in the OEC results in impaired kinetics of state transitions (Allahverdiyeva et al. 2013). It is important to point out that the overall protein abundances of the proteins, which we found differentially accumulated in the PSI complexes and PSII dimers (PSI/PSII) complex and LHCII trimer in *nsi* were not found altered in their total protein abundance compared to wt (see Supplemental dataset 2; Koskela et al. 2018). Hence, we hypothesize that chemical modifications of these proteins, such as lysine acetylation, might alter their ability to engage in protein–protein interactions and to reside in specific complexes.

Taken together, our results imply that despite the major molecular phenotype (lack of interaction between PSI and LHCII and thus state transitions) in the *nsi* and *stn7* mutants, only minor changes could be detected in the composition of the involved protein complexes. Therefore, different kinds of post-translational modifications, such as acetylation and phosphorylation, appear to control the dynamic structure of thylakoid membrane and to regulate the formation of protein complexes in response to environmental cues. Future studies will reveal the impact of OEC on complex formation and resolve the putative role of OHP1 in the *nsi* mutants.

## Electronic supplementary material

Below is the link to the electronic supplementary material.

Figure S1. 2D-Blue Native gels of wt and nsi-1 thylakoid protein complexes. Thylakoid samples from growth light (100 µmol photons m-2 s-1) adapted plants were solubilized either with (a) 1% (w/v) digitonin (5 µg of chlorophyll) or (b) 1% (w/v) dodecyl maltoside (3.5 μg of chlorophyll) prior to lpBN gel electrophoresis. lp-Blue Native PAGE was followed by separation of protein complexes in the second dimension on 12% reducing SDS-PAGE supplemented with 6 M urea and SYPRO staining. The proteins were identified based on Aro et al. 2005 and Suorsa et al. 2015. sc, supercomplex; PSIId, PSII dimer; PSIIm, PSII monomer; Cytb6f, cytochrome b6f. Supplementary file1 (TIF 52959 kb)

Table S1. List of all proteins identified and quantified, and list of lysine-acetylated proteins within the PSI/PSII (Table S1A, Table S1C) and LHCII trimer (Table S1B, Table S1D) bands excised from the lpBN gel. Thylakoid membranes were solubilized with digitonin, complexes separated using lpBN gel electrophoresis and selected bands excised for LC-MS/MS analysis. LC-MS/MS raw data were processed using MaxQuant software (version 1.5.2.8, https://www.maxquant.org/; Cox and Mann, 2008) and the Araport 11 database as described in material and methods. Selection criteria for identifications: peptide and protein FDR < 1%, contaminant non-plant proteins and non-plastid proteins (according to Suba3 con) were removed. Most abundant proteins within one genotype can be estimated by their iBAQ values. iBAQ: intensity-based absolute quantification as described by Schwanhäusser et al. (2011). Three biological replicates (Rep1-3) per genotypes were analysed. Supplementary file2 (XLSX 471 kb)

Table S2. List of all proteins identified and quantified, and list of lysine-acetylated proteins within the PSI/PSII (Table S2A, S2C) and LHCII trimer (Table S2B, S2D) bands excised from the lpBN gel. Thylakoid membranes were solubilized with dodecyl maltoside, complexes separated using lpBN gel electrophoresis and selected bands excised for LC-MS/MS analysis. LC-MS/MS raw data were processed using MaxQuant software (version 1.5.2.8, https://www.maxquant.org/; Cox and Mann, 2008) and the Araport 11 database as described in material and methods. Selection criteria for identifications: peptide and protein FDR < 1%, contaminant non-plant proteins and non-plastid proteins (according to Suba3 con) were removed. Most abundant proteins within one genotype can be estimated by their iBAQ values. iBAQ: intensity-based absolute quantification as described by Schwanhäusser et al. (2011). Three biological replicates (Rep1-3) per genotypes were analysed. Supplementary file3 (XLSX 540 kb)

Tables S3–S7. P-values of the statistical tests performed to compare the relative protein abundances presented in tables 1–4. Supplementary file4 (DOCX 38 kb)
