# Supplementary figures and images for: Comparative analysis of thylakoid protein complexes in state transition mutants nsi and stn7: focus on PSI and LHCII
Source: Photosynth Res. 2020 Jan 23;145(1):15–30. doi: 10.1007/s11120-020-00711-4 (PMC7308260; doi:10.1007/s11120-020-00711-4)

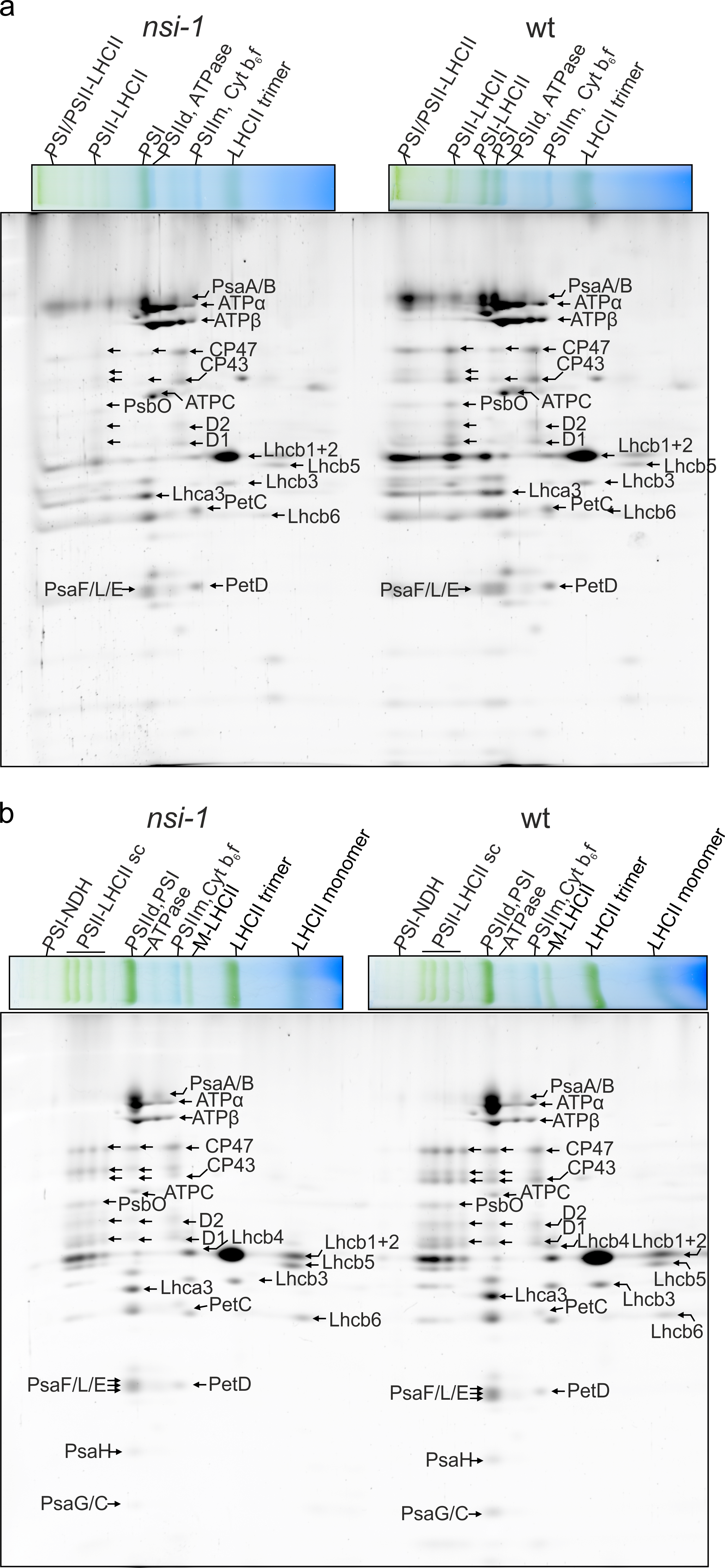

Supplement: Supplementary file 1 — Figure S1. 2D-Blue Native gels of wt and nsi-1 thylakoid protein complexes. Thylakoid samples from growth light (100 µmol photons m-2 s-1) adapted plants were solubilized either with (a) 1% (w/v) digitonin (5 µg of chlorophyll) or (b) 1% (w/v) dodecyl maltoside (3.5 μg of chlorophyll) prior to lpBN gel electrophoresis. lp-Blue Native PAGE was followed by separation of protein complexes in the second dimension on 12% reducing SDS-PAGE supplemented with 6 M urea and SYPRO staining. The proteins were identified based on Aro et al. 2005 and Suorsa et al. 2015. sc, supercomplex; PSIId, PSII dimer; PSIIm, PSII monomer; Cytb6f, cytochrome b6f. Supplementary file1 (TIF 52959 kb) [file 11120_2020_711_MOESM1_ESM.tif]
