# Supplementary material for: Comparative analysis of thylakoid protein complexes in state transition mutants nsi and stn7: focus on PSI and LHCII
Source: Photosynth Res. 2020 Jan 23;145(1):15–30. doi: 10.1007/s11120-020-00711-4 (PMC7308260; doi:10.1007/s11120-020-00711-4)
Supplement: Supplementary file 4 — Tables S3–S7. P-values of the statistical tests performed to compare the relative protein abundances presented in tables 1–4. Supplementary file4 (DOCX 38 kb) [file 11120_2020_711_MOESM4_ESM.docx]

**Table S3.** Homogeneity of variance -test and ANOVA p-values for digitonin solubilized PSI/PSII-dimer -complex proteins. Homogeneity of variance was tested with Levene’s test. Difference in protein abundance between genotypes was tested using ANOVA. If variances were not homogenous (Levene’s p < 0.05), Brown-Forsythe -test was used instead. P-values below 0.05 were considered as significant (green highlight). n/a = not applicable.

| **Proteins/protein groups** | | **Levene’s test p-value** | **ANOVA p-value** | **Brown-Forsythe p-value** |
| --- | --- | --- | --- | --- |
| **Locus/loci** | **Name(s)** |  |  |  |
| AT4G28750 | PSAE-1 | 0.043 | n/a | 0.864 |
| AT4G02770 | PSAD-1 | 0.031 | n/a | 0.779 |
| AT1G55670 | PSAG | 0.45 | 0.386 | n/a |
| ATCG01060 | PSAC | 0.707 | 0.978 | n/a |
| ATCG00350 | PSAA | n/a | n/a | n/a |
| AT1G31330 | PSAF | 0.382 | 0.861 | n/a |
| AT1G61520 | LHCA3 | 0.195 | 0.505 | n/a |
| AT3G54890 | LHCA1 | 0.541 | 0.945 | n/a |
| AT1G52230 | PSAH-2 | 0.372 | 0.518 | n/a |
| AT3G47470 | LHCA4 | 0.072 | 0.407 | n/a |
| ATCG00340 | PSAB | 0.306 | 0.413 | n/a |
| AT4G12800 | PSAL | 0.442 | 0.155 | n/a |
| AT1G30380 | PSAK | 0.093 | 0.771 | n/a |
| AT3G61470; AT5G28450 | LHCA2;  CAB family | 0.166 | 0.176 | n/a |
| AT2G20260 | PSAE-2 | 0.058 | 0.949 | n/a |
| AT5G64040 | PSAN | 0.034 | n/a | 0.725 |
| AT2G46820 | PSAP | 0.337 | 0.682 | n/a |
| AT1G08380 | PSAO | 0.771 | 0.699 | n/a |
| ATCG00280 | CP43 (PSBC) | 0.026 | n/a | 0.188 |
| AT1G29920; AT1G29910 | LHCB1.1;  LHCB1.2 | 0.817 | 0.021 | n/a |
| ATCG00680 | CP47 (PSBB) | 0.070 | 0.120 | n/a |
| ATCG00270 | D2 (PSBD) | 0.114 | 0.065 | n/a |
| ATCG00020 | D1 (PSBA) | 0.025 | n/a | 0.318 |
| AT4G10340 | CP26 (LHCB5) | 0.155 | 0.794 | n/a |
| AT3G50820 | PSBO-2 | 0.178 | 0.003 | n/a |
| AT3G08940 | CP29-2 (LHCB4.2) | 0.007 | 0.197 | n/a |
| AT1G15820 | CP24 (LHCB6) | 0.057 | 0.412 | n/a |
| AT3G27690; AT2G05070; AT2G05100 | LHCB2.4; LHCB2.2;  LHCB2.1 | 0.184 | 0.361 | n/a |
| ATCG00560 | PSBL | 0.206 | 0.072 | n/a |
| AT5G66570 | PSBO-1 | 0.522 | 0.031 | n/a |
| AT5G01530 | CP29-1 (LHCB4.1) | 0.193 | 0.346 | n/a |
| AT2G34420 | LHCB1.5 | 0.085 | 0.957 | n/a |
| AT5G54270 | LHCB3 | 0.183 | 0.759 | n/a |

**Table S4.** Homogeneity of variance -test and ANOVA p-values for DM solubilized PSI/PSII-dimer -complex proteins. Homogeneity of variance was tested with Levene’s test. Differences in protein abundances between genotypes were tested using ANOVA or, if variances were not homogenous (Levene’s p < 0.05), the Brown-Forsythe -test was used instead. P-values below 0.05 were considered as significant (green highlight). n/a = not applicable.

| **Proteins/protein groups** | | **Levene’s test p-value** | **ANOVA p-value** | **Brown-Forsythe p-value** |
| --- | --- | --- | --- | --- |
| **Locus/loci** | **Name(s)** |  |  |  |
| AT4G02770 | PSAD-1 | 0.319 | 0.385 | n/a |
| AT4G28750 | PSAE-1 | 0.365 | 0.564 | n/a |
| ATCG01060 | PSAC | 0.100 | 0.704 | n/a |
| AT1G55670 | PSAG | 0.266 | 0.636 | n/a |
| ATCG00350 | PSAA | n/a | n/a | n/a |
| AT3G47470 | LHCA4 | 0.232 | 0.483 | n/a |
| AT1G61520 | LHCA3 | 0.738 | 0.814 | n/a |
| AT1G31330 | PSAF | 0.045 | n/a | 0.549 |
| AT3G54890 | LHCA1 | 0.418 | 0.855 | n/a |
| ATCG00340 | PSAB | 0.506 | 0.859 | n/a |
| AT1G52230 | PSAH-2 | 0.366 | 0.983 | n/a |
| AT3G61470 | LHCA2 | 0.692 | 0.595 | n/a |
| AT4G12800 | PSAL | 0.973 | 0.879 | n/a |
| AT1G30380 | PSAK | 0.027 | n/a | 0.404 |
| AT2G20260 | PSAE-2 | 0.171 | 0.634 | n/a |
| AT5G64040 | PSAN | 0.957 | 0.965 | n/a |
| AT1G08380 | PSAO | 0.025 | n/a | 0.512 |
| ATCG00680 | CP47 (PSBB) | 0.495 | 0.274 | n/a |
| ATCG00280 | CP43 (PSBC) | 0.283 | 0.019 | n/a |
| ATCG00270 | D2 (PSBD) | 0.032 | n/a | 0.154 |
| ATCG00020 | D1 (PSBA) | 0.031 | n/a | 0.194 |
| AT5G66570 | PSBO-1 | 0.111 | 0.061 | n/a |
| AT1G29930; AT1G29920; AT1G29910 | LHCB1.3; LHCB1.1; LHCB1.2 | 0.225 | 0.895 | n/a |
| AT4G10340 | CP26 (LHCB5) | 0.014 | n/a | 0.684 |
| ATCG00560 | PSBL | 0.001 | n/a | 0.097 |
| AT3G08940 | CP29.2 (LHCB4.2) | 0.002 | n/a | 0.571 |
| AT3G50820 | PSBO-2 | 0.096 | 0.032 | n/a |
| AT2G05070 | LHCB2.2 | 0.575 | 0.518 | n/a |
| AT2G34430 | LHCB1.4 | 0.631 | 0.593 | n/a |

**Table S5.** Homogeneity of variance test and ANOVA p-values for digitonin solubilized LHCII-complex proteins. Homogeneity of variance was tested with Levene’s test. Difference in protein abundance between genotypes was tested using ANOVA. If variances were not homogenous (Levene’s p < 0.05), Brown-Forsythe -test was used instead. P-values below 0.05 were considered as significant (green highlight). n/a = not applicable.

| **Proteins/protein groups** | | **Levene’s test p-value** | **ANOVA p-value** | **Brown-Forsythe p-value** |
| --- | --- | --- | --- | --- |
| **Locus/loci** | **Name(s)** |  |  |  |
| AT2G05070 | LHCB2.2 | 0.012 | n/a | 0.592 |
| AT2G34420 | LHCB1.5 | 0.362 | 0.366 | n/a |
| AT5G54270 | LHCB3 | n/a | n/a | n/a |
| AT2G34430 | LHCB1.4 | 0.117 | 0.127 | n/a |
| ATCG00680 | CP47 (PSBB) | 0.039 | n/a | 0.173 |
| ATCG00020 | D1 (PSBA) | 0.302 | 0.140 | n/a |
| ATCG00270 | D2 (PSBD) | 0.040 | n/a | 0.093 |
| AT1G44575 | NPQ4 (PSBS) | 0.102 | 0.146 | n/a |
| AT1G34000 | OHP2 | 0.064 | 0.083 | n/a |
| AT4G10340 | CP26 (LHCB5) | 0.293 | 0.215 | n/a |
| AT5G64040 | PSAN | 0.168 | 0.068 | n/a |
| AT5G02120 | OHP1 | 0.542 | 0.027 | n/a |
| AT1G29930 | LHCB1.3 | 0.041 | n/a | 0.362 |
| AT3G47470 | LHCA4 | 0.002 | n/a | 0.589 |
| ATCG00280 | CP43 (PSBC) | 0.119 | 0.013 | n/a |
| AT3G54890 | LHCA1 | 0.003 | n/a | 0.489 |
| AT1G31330 | PSAF | 0.022 | n/a | 0.486 |
| AT1G61520 | LHCA3 | 0.024 | n/a | 0.201 |
| AT1G52230 | PSAH-2 | 0.011 | n/a | 0.586 |
| AT2G46820 | PSAP | 0.020 | n/a | 0.061 |
| AT1G79040 | PSAR | 0.017 | n/a | 0.334 |
| AT3G08940 | CP29-2 (LHCB4.2) | 0.530 | 0.207 | n/a |
| AT5G66570 | PSBO-1 | 0.546 | 0.519 | n/a |
| AT3G61470; AT5G28450 | LHCA2;  CAB family | 0.011 | n/a | 0.476 |
| ATCG00340 | PSAB | 0.008 | n/a | 0.414 |
| AT1G15820 | CP24 (LHCB6) | 0.703 | 0.659 | n/a |
| AT4G28750 | PSAE-1 | 0.072 | 0.229 | n/a |
| ATCG00350 | PSAA | 0.008 | n/a | 0.319 |
| AT5G51545 | LPA2 | 0.688 | 0.182 | n/a |
| AT5G01530 | CP29-1 (LHCB4.1) | 0.907 | 0.088 | n/a |
| AT4G12800 | PSAL | 0.013 | n/a | 0.466 |
| AT4G02770 | PSAD-1 | 0.005 | n/a | 0.417 |
| AT1G03600 | PSB27 | 0.051 | 0.232 | n/a |
| ATCG00560 | PSBL | 0.004 | n/a | 0.684 |
| AT1G08380 | PSAO | n/a | 0.807 | n/a |
| AT1G30380 | PSAK | 0.002 | n/a | 0.597 |

**Table S6.** Homogeneity of variance test and ANOVA p-values for DM solubilized LHCII-dimer -complex proteins. Homogeneity of variance was tested with Levene’s test. Difference in protein abundance between genotypes was tested using ANOVA. If variances were not homogenous (Levene’s p < 0.05), Brown-Forsythe -test was used instead. P-values below 0.05 were considered as significant (green highlight). n/a = not applicable.

| **Proteins/protein groups** | | **Levene’s test p-value** | **ANOVA p-value** | **Brown-Forsythe p-value** |
| --- | --- | --- | --- | --- |
| **Locus/loci** | **Name(s)** |  |  |  |
| AT2G05070 | LHCB2.2 | 0.038 | n/a | 0.327 |
| AT2G34430 | LHCB1.4 | 0.299 | 0.097 | n/a |
| AT2G34420 | LHCB1.5 | 0.105 | 0.267 | n/a |
| AT1G29920; AT1G29910 | LHCB1.1; LHCB1.2 | 0.569 | 0.555 | n/a |
| AT5G54270 | LHCB3 | n/a | n/a | n/a |
| AT4G10340 | CP26 (LHCB5) | 0.080 | 0.604 | n/a |
| AT3G08940 | CP29.2 (LHCB4.2) | 0.003 | n/a | 0.552 |
| ATCG00680 | CP47 (PSBB) | 0.042 | n/a | 0.311 |
| ATCG00270 | D2 (PSBD) | 0.564 | 0.675 | n/a |
| AT3G16140 | PSAH-1 | 0.002 | n/a | 0.567 |
| ATCG00020 | D1 (PSBA) | 0.021 | n/a | 0.420 |
| AT5G66570 | PSBO-1 | 0.438 | 0.685 | n/a |
| AT3G47470 | LHCA4 | 0.006 | n/a | 0.584 |
| AT1G44575 | NPQ4 (PSBS) | 0.757 | 0.784 | n/a |
| AT5G01530 | CP29.1 (LHCB4.1) | 0.010 | n/a | 0.577 |
| AT1G15820 | CP24 (LHCB6) | 0.056 | 0.809 | n/a |
| AT1G31330 | PSAF | 0.104 | 0.995 | n/a |
| ATCG00340 | PSAB | 0.009 | n/a | 0.593 |
| AT5G47110 | LIL3.2 | 0.451 | 0.894 | n/a |
| AT4G17600 | LIL3.1 | 0.976 | 0.623 | n/a |
| AT1G61520 | LHCA3 | 0.130 | 0.715 | n/a |
| AT1G34000 | OHP2 | 0.168 | 0.081 | n/a |
| ATCG00280 | CP43 (PSBC) | 0.045 | n/a | 0.329 |
| AT3G54890 | LHCA1 | 0.046 | n/a | 0.693 |
| AT4G28750 | PSAE-1 | 0.507 | 0.651 | n/a |
| AT3G61470 | LHCA2 | 0.010 | n/a | 0.678 |
| AT5G02120 | OHP1 | 0.853 | 0.314 | n/a |
| AT4G12800 | PSAL | 0.008 | n/a | 0.524 |
| AT5G64040 | PSAN | 0.124 | 0.212 | n/a |
| ATCG00350 | PSAA | 0.005 | n/a | 0.542 |
| AT1G30380 | PSAK | 0.003 | n/a | 0.546 |
| AT4G02770 | PSAD-1 | 0.005 | n/a | 0.530 |
| AT1G79040 | PSBR | 0.114 | 0.878 | n/a |
| AT1G52230 | PSAH-2 | 0.021 | n/a | 0.477 |
| AT1G08380 | PSAO | n/a | n/a | n/a |
| AT3G50820 | PSAO-2 | 0.733 | 0.195 | n/a |
| AT4G21280 | PSBQ-1 | 0.047 | n/a | 0.224 |

**Table S7**. Multiple comparisons of significantly different protein groups (ANOVA p < 0.05) between genotypes using Tukey-HSD. Each protein is presented as an individual table with p-values of the multiple comparisons. Differences with p < 0.05 were considered significant (green). n/a = not applicable.

**DIGITONIN, PSI COMPLEX**

| **LHCB1.1; LHCB1.2** | **p-value** |
| --- | --- |
| wt vs. *nsi-1* | 0.142 |
| wt vs. *nsi-2* | 0.045 |
| wt vs. *stn7* | 0.019 |
| *nsi-1* vs. *nsi-2* | 0.846 |
| *nsi-1* vs. *stn7* | 0.515 |
| *nsi-2* vs. *stn7* | 0.924 |

| **PSBO-2** | **p-value** |
| --- | --- |
| wt vs. *nsi-1* | 0.011 |
| wt vs. *nsi-2* | 0.004 |
| wt vs. *stn7* | 0.374 |
| *nsi-1* vs. *nsi-2* | 0.897 |
| *nsi-1* vs. *stn7* | 0.114 |
| *nsi-2* vs. *stn7* | 0.043 |

| **PSBO-1** | **p-value** |
| --- | --- |
| wt vs. *nsi-1* | 0.054 |
| wt vs. *nsi-2* | 0.075 |
| wt vs. *stn7* | 0.889 |
| *nsi-1* vs. *nsi-2* | 0.995 |
| *nsi-1* vs. *stn7* | 0.147 |
| *nsi-2* vs. *stn7* | 0.203 |

**DM, PSI/PSII-DIMER -COMPLEX**

| **CP43** | **p-value** | |
| --- | --- | --- |
| wt vs. *nsi-1* | 0.278 | |
| wt vs. *nsi-2* | 0.077 | |
| wt vs. *stn7* | 0.822 | |
| *nsi-1* vs. *nsi-2* | 0.787 | |
| *nsi-1* vs. *stn7* | 0.085 | |
| *nsi-2* vs. *stn7* | 0.023 | |
|  | |  |
| **PSBO-2** | **p-value** | |
| wt vs. *nsi-1* | 0.959 | |
| wt vs. *nsi-2* | 0.945 | |
| wt vs. *stn7* | 0.095 | |
| *nsi-1* vs. *nsi-2* | 1.000 | |
| *nsi-1* vs. *stn7* | 0.048 | |
| *nsi-2* vs. *stn7* | 0.044 | |

**DIGITONIN, LHCII-TRIMER**

| **OHP1** | **p-value** |
| --- | --- |
| wt vs. *nsi-1* | 0.056 |
| wt vs. *nsi-2* | 0.025 |
| wt vs. *stn7* | 0.203 |
| *nsi-1* vs. *nsi-2* | 0.936 |
| *nsi-1* vs. *stn7* | 0.796 |
| *nsi-2* vs. *stn7* | 0.482 |

| **CP43** | **p-value** |
| --- | --- |
| wt vs. *nsi-1* | 0.043 |
| wt vs. *nsi-2* | 0.010 |
| wt vs. *stn7* | 0.117 |
| *nsi-1* vs. *nsi-2* | 0.726 |
| *nsi-1* vs. *stn7* | 0.890 |
| *nsi-2* vs. *stn7* | 0.355 |
